# Characterisation of L-Type Amino Acid Transporter 1 (LAT1) Expression in Human Skeletal Muscle by Immunofluorescent Microscopy

**DOI:** 10.3390/nu10010023

**Published:** 2017-12-26

**Authors:** Nathan Hodson, Thomas Brown, Sophie Joanisse, Nick Aguirre, Daniel W. D. West, Daniel R. Moore, Keith Baar, Leigh Breen, Andrew Philp

**Affiliations:** 1 School of Sport, Exercise and Rehabilitation Sciences, University of Birmingham, Birmingham B15 2TT, UK; NWH151@student.bham.ac.uk (N.H.); txb208@alumni.bham.ac.uk (T.B.); S.Joanisse@bham.ac.uk (S.J.); l.breen@bham.ac.uk (L.B.); 2 University of California Davis, Davis, CA 95616, USA; nwaguirre@ucdavis.edu (N.A.); kbaar@ucdavis.edu (K.B.); 3 Faculty of Kinesiology and Physical Education, University of Toronto, Toronto, ON M5S 1A1, Canada; daniel.west@utoronto.ca (D.W.D.W.); dr.moore@utoronto.ca (D.R.M.)

**Keywords:** LAT1, leucine, protein, amino acid transport

## Abstract

The branch chain amino acid leucine is a potent stimulator of protein synthesis in skeletal muscle. Leucine rapidly enters the cell via the L-Type Amino Acid Transporter 1 (LAT1); however, little is known regarding the localisation and distribution of this transporter in human skeletal muscle. Therefore, we applied immunofluorescence staining approaches to visualise LAT1 in wild type (WT) and LAT1 muscle-specific knockout (mKO) mice, in addition to basal human skeletal muscle samples. LAT1 positive staining was visually greater in WT muscles compared to mKO muscle. In human skeletal muscle, positive LAT1 staining was noted close to the sarcolemmal membrane (dystrophin positive staining), with a greater staining intensity for LAT1 observed in the sarcoplasmic regions of type II fibres (those not stained positively for myosin heavy-chain 1, Type II—25.07 ± 5.93, Type I—13.71 ± 1.98, *p* < 0.01), suggesting a greater abundance of this protein in these fibres. Finally, we observed association with LAT1 and endothelial nitric oxide synthase (eNOS), suggesting LAT1 association close to the microvasculature. This is the first study to visualise the distribution and localisation of LAT1 in human skeletal muscle. As such, this approach provides a validated experimental platform to study the role and regulation of LAT1 in human skeletal muscle in response to various physiological and pathophysiological models.

## 1. Introduction

Amino acid transport is an essential component in the survival of all cells [1], providing substrates for processes such as protein synthesis [2,3] and cell division [4]. Amino acid transport into cells is of particular importance within skeletal muscle to maintain positive protein balance [2,3]. Some evidence suggests that amino acid transporter proteins may also act as receptors, converting extracellular amino acid abundance into intracellular signals [5,6,7], a role termed ‘transceptor’ [8].

Of the amino acid transporters identified in skeletal muscle, the L-Type amino acid transporter 1 (LAT1) also known as the solute carrier family 7 member 5 (SLC7A5) has received significant attention [9,10,11,12,13,14,15] due to its role in the ingress of amino acids, vital for optimal stimulation of muscle protein synthesis (MPS) [16,17,18]. LAT1 transports leucine (or other branched chain amino acids (BCAAs)) into the cell via a bi-transport system that simultaneously exports glutamine [19]. The increase in intracellular leucine/BCAAs subsequently activates the mechanistic target of rapamycin (mTOR) complex 1 (mTORC1), resulting in an elevation in MPS [20]. Leucine’s reported potency toward mTORC1 in skeletal muscle [16] is modulated primarily through LAT1, as this is the main transporter by which leucine moves into cells [9]. Other L-type amino acid transporters are expressed in skeletal muscle, including LAT2 and LAT4 [21,22]; however, it seems that LAT1 is the primary BCAA transporter. In LAT1 muscle-specific knockout (mKO) mice, a compensatory increase in SLC7A8/LAT2 mRNA was reported [14], yet a significant reduction in amino acid transport into muscle was apparent, suggesting that LAT1 is integral to this process. Furthermore, LAT4 is only weakly expressed in skeletal muscle [21] and is believed to be primarily required in placenta as this transporter functions to deliver amino acids from the mother to the foetus [23]. Due to its apparent importance to amino acid transport in skeletal muscle, understanding the role and regulation of LAT1 is essential for studies aimed at maximising the delivery of dietary protein and amino acids in the context of post-exercise recovery.

Gene expression and protein content of LAT1 are positively associated with mTORC1 activity; however, increases in LAT1 expression are abolished by the administration of rapamycin, suggesting that mTORC1 may also regulate LAT1 activity [24]. In human skeletal muscle, essential AA (EAA) consumption elicits an early increase in LAT1 gene expression followed by elevations in protein content [12]. Furthermore, these transient effects have also been reported in response to resistance exercise alone [11], and in combination with EAA consumption [10]. Such data suggest that anabolic stimuli may initiate signalling pathways, which upregulate both LAT1 gene expression and protein levels in human skeletal muscle, possibly translating to greater leucine/BCAA transport and a superior protein synthetic response [2,3,16,25]. However, it is difficult to reconcile the physiological relevance of changes in LAT1 gene and protein content with LAT1 activity since LAT1 is only active when at the plasma membrane [9,26]. Therefore, any measure of protein or gene expression in whole muscle homogenates [10,11,12] may not allow reliable conclusions to be drawn. To date, the localisation of LAT1 in human muscle is unknown and requires investigation.

Therefore, the aim of the present study was develop and validate immunofluorescent approaches to study LAT1 cellular distribution and localisation in human skeletal muscle. We hypothesised that, in basal human skeletal muscle, LAT1 would be located at the plasma membrane and would be ubiquitously expressed across fibre types.

## 2. Materials and Methods 

### 2.1. Mouse Skeletal Muscle Sample Collection and Preparation

LAT1 mKO mice were provided as a gift by Dr. Peter Taylor of the University of Dundee. Methods of generation of this mouse strain have been previously described [14]. Mice (*n* = 2 mKO, *n* = 2 wild type (WT)) were housed in a climate-controlled facility at the University of California Davis, in a standard 12 h light/dark cycle and fed standard chow ad libitum. All procedures were approved by the UC Davis Institutional Animal Care and Use Committee (IACUC) and performed under protocol number 19244. At three months of age, mice were fasted for 5 h before the extensor digitorum longus (EDL) muscle was surgically removed under anaesthetic (2.5% isofluorane), pinned to cork and frozen in liquid nitrogen-cooled isopentane. Muscles were subsequently stored at −80 °C until sectioned. Prior to sectioning, the belly of the EDL was blocked in Tissue-Tek Optimal Cutting Temperature (OCT) Compound (VWR International, Leicestershire, UK) and frozen in liquid nitrogen-cooled isopentane, at which point the sample was ready for cryo-sectioning. During staining protocols, both WT and mKO samples were processed and stained simultaneously, and image capture paradigms were also identical.

### 2.2. Human Skeletal Muscle Sample Collection and Preparation

Ethical approval for the collection of muscle samples was granted by the NHS West Midlands Black Country Research Ethics Committee (14/WM/0088 and 15/WM/0003), the Hamilton Health Sciences Research Ethics Board (12-631), and the University of Guelph Research Ethics Board (120C018). The study conformed to the standards presented by the Declaration of Helsinki (seventh version). Written informed consent was obtained from all participants before samples were collected. Muscle samples for immunofluorescence were obtained from the vastus lateralis muscle of 6 young, healthy males (mean age—22 ± 2 years), who attended the laboratory after an overnight (10 h) fast, using the Bergstrom percutaneous needle technique. Samples were blotted free of excess blood and dissected free of any fat or connective tissue before being placed in OCT compound and frozen in liquid nitrogen-cooled isopentane. Muscle samples were then stored at −80 °C until further analysis. Muscle samples for Western blot were collected after an overnight fast and 15 min after an acute bout of exercise, as previously described [27]. Samples (200–300 mg) were immediately processed for sarcolemmal vesicles and cytosolic fractions, as previously described [28], before being stored at −80 °C until further analysis.

### 2.3. Immunofluorescent Staining

Embedded muscle samples were fixed in front of the microtome blade (Bright 5040, Bright Instrument Company Limited, Huntingdon, UK) and cryo-sections (5 μm) collected onto room temperature uncoated glass slides (VWR International, Lutterworth, UK). Sections were left to air dry at room temperature for 10 min to remove excess crystallized water inside sections under storage. Sections were fixed in acetone and ethanol (3:1) solution (Fisher, Loughborough, UK) for 5 min and then washed for 3 × 5 min in phosphate buffered saline (PBS) supplemented with 0.2% Tween (PBS-T) to remove fixation reagent. Sections were then incubated in primary antibody solution diluted with 5% normal goat serum (Invitrogen, Loughborough, UK) for 2 h at room temperature. For blocking peptide experiments, the anti-SLC7A5 antibody was pre-incubated with its corresponding peptide (20 times greater than primary antibody concentration) on an orbital shaker for 1 h at room temperature. Following incubation, sections were washed for 3 × 5 min in PBS-T and incubated in the appropriate secondary antibody for 1 h at room temperature. If needed, sections were finally incubated with Wheat Germ Agglutinin (WGA-350) for 30 min at room temperature to mark the sarcolemmal membrane. After a final wash in PBS, slides were left to air dry until the visual water stains evaporated: 1–2 min at room temperature. Sections were mounted with 20 μL Mowiol^®^ 4-88 (Sigma-Aldrich, Gillingham, UK) and sealed by glass coverslips to protect the muscle sections and to preserve fluorescence signals. Slides were left overnight in a dark room before observation. Primary antibodies, blocking peptides and corresponding secondary antibodies and working dilutions are listed in Table 1. All secondary antibodies were sourced from ThermoFisher Scientific Inc. (Waltham, MA, USA).

### 2.4. Image Capture

Prepared slides were observed under a Nikon E600 widefield microscope using a 40 × 0.75 numerical aperture objective. Images per area were captured under three colour filters achieved by a SPOT RT KE colour three shot CCD camera (Diagnostic Instruments Inc., Sterling Heights, MI, USA), illuminated by a 170 W Xenon light source. For image capture, 4′,6-diamidino-2-phenylindole (DAPI) UV (340–380 nm) filter was used to view WGA-350 (blue) signals and LAT1 stains tagged with Alexa 488 fluorophores (green) were visualised under the Fluorescein isothiocyanate (FITC) (465–495 nm) excitation filter. The Texas-Red (540–580 nm) excitation filter was used to capture signals of eNOS (blood vessels), dystrophin (sarcolemma) or Myosin heavy chain 1 (Type I Fibres), which were conjugated with Alexa Fluor 594 fluorophores. On average, 8 images were captured per section, and each image contained ~8 muscle fibres. As 2 sections per participant were imaged, approximately 120 fibres per subject were used for analysis. Image processing and analysis was undertaken on ImagePro Plus 5.1 (Media Cybernetics, Rockville, MD, USA) and all factors, i.e., exposure time and gain, were kept constant between all images on each individual slide. All images underwent flattening and de-speckling prior to quantification of co-localization to limit the contribution of background, non-specific fluorescence. Image signals generated by WGA or dystrophin were used to estimate cell membrane borders, and MHC1 staining was utilised to identify type I fibres. The intensity of LAT1 staining in these groups of fibres was used to assess any differences in fibre type abundance of the LAT1 protein.

### 2.5. Immunoblotting

As a qualitative methodological comparator with immunofluorescence for the spatial distribution of LAT1, giant sarcolemmal vesicles were isolated from the vastus lateralis of a subset healthy males (*n* = 2, ~21 years of age) as previously described [29]. The protein pellets that remained were resuspended and homogenized in a buffer containing 3-(*N*-morpholino)propanesulfonic acid (MOPS) (10 mM), KCl (140 mM), Ethylenediaminetetraacetic acid (EDTA) (10 mM), and protease inhibitors. Samples were centrifuged at 800× *g* 10 min at 4 °C to remove insoluble proteins. Protein concentrations in cytosolic and sarcolemmal vesicle fractions were determined by BCA assay and prepared at equal concentrations in 4× Laemmli buffer. In addition, 3 µg of protein were loaded on a precast gel (BioRad 5671094), run at 200 V for 45 min, transferred (100 V for 1 h) to nitrocellulose membrane, stained with Ponceau S solution, blocked in 5% skim milk, and probed overnight (1:1000 in Tris-buffered saline, supplemented with 0.1% Tween (TBST)) using the same anti-LAT1 antibody used for immuno-fluorescent staining (see Table 1). The membrane was incubated in goat anti-rabbit IgG Horseradish peroxidase (HRP) conjugated secondary (Thermo Fisher, cat. 31460; 1:10,000 in TBST) for 1 h at RT before washing in TBST and detection by chemiluminescence. Images were captured using a Fluorochem E Imaging system (Protein Simple; Alpha Innotech, Santa Clara, CA, USA).

### 2.6. Statistical Analysis 

All statistical analysis was conducted on SPSS version 22 for Windows (SPSS Inc., Chicago, IL, USA). Differences between the staining intensity in type I and type II fibres was analysed by a paired-samples *t*-test. Significance threshold was set at *p* ≤ 0.05. Data are presented as Mean ± SE unless otherwise stated.

## 3. Results

### 3.1. Characterisation and Validation of LAT1 Immunofluorescence Labelling Approaches

**Figure 1:**
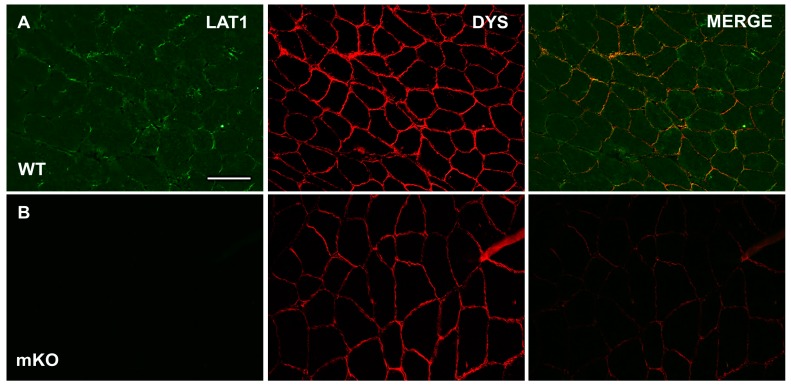
Immunofluorescent detection of L-Type amino acid transporter 1 (LAT1) in wild type (WT) and LAT1 muscle-specific knockout (mKO) mouse extensor digitorum longus (EDL) muscle. Sections were stained with anti-LAT1 antibody (far left panels, green) and then co-stained with dystrophin (middle panels, red) for the identification of the sarcolemma. Representative images for both WT (**A**) and mKO (**B**) are shown. Scale bar in top left panel is 50 µm and all images were captured at the same magnification.

In WT skeletal muscle samples, positive LAT1 signals were noted at the periphery of many fibres, in addition to displaying a stronger sarcoplasmic staining than seen in mKO samples (Figure 1A). In mKO samples, positive LAT1 staining was almost completely abolished (Figure 1B). We take this data to suggest that the anti-LAT1 antibody utilised here is specific to the LAT1 protein in skeletal muscle, This was further confirmed, as the pattern of staining noted in mKO samples was similar to that seen when the primary antibody was omitted from staining protocols.

**Figure 2:**
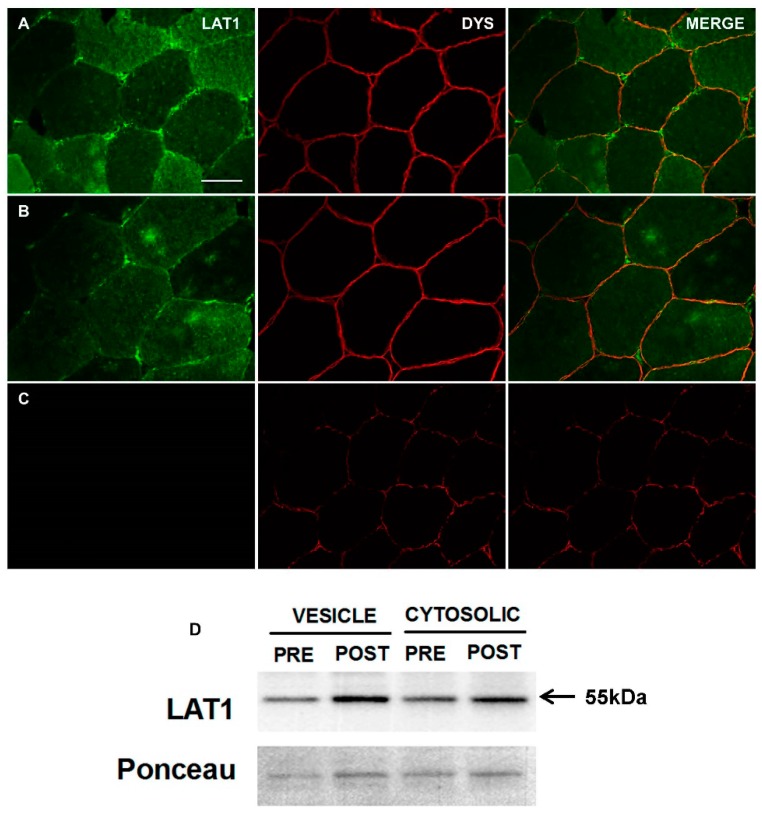
Immunofluorescent detection of LAT1 in human vastus lateralis muscle. Sections were stained with anti-LAT1 antibody (far left panels, green) and then co-stained with dystrophin (middle panels, red) for the identification of the sarcolemma. Representative images from two participants are displayed (**A**,**B**). Use of a peptide competition assay reduced the signal intensity of LAT1 staining (**C**). A strong positive signal was noted close to the sarcolemmal membrane and possible sites of blood vessels. In addition, a possible fibre type difference in LAT1 staining was noted. Immunoblots for the LAT1 protein displayed bands to be apparent in both giant sarcolemmal vesicles and cytosolic fractions before (PRE) and after (POST) an acute bout of resistance exercise (**D**) (*n* = 2). Scale bar in top left panel is 50 µm and all images were captured at the same magnification.

Next, we applied the same staining technique to human skeletal muscle (Figure 2A,B). Here, a similar staining pattern was noted, with positive-LAT1 puncta close to the sarcolemmal membrane (dystrophin positive staining). Additionally, a possible fibre-type difference in LAT1 content was apparent in human tissue. Finally, when the anti-LAT1 antibody was incubated with a LAT1-specific blocking peptide, the immunofluorescent signal and staining pattern was greatly reduced (Figure 2C), further confirming the antibody’s specificity to the LAT1 protein. The immunoblot data also demonstrate the presence of LAT1 in the sarcolemma and cytosolic protein fractions (Figure 2D).

### 3.2. LAT1 Protein Content Is Greater in Fast-Twitch Skeletal Muscle Fibres

**Figure 3:**
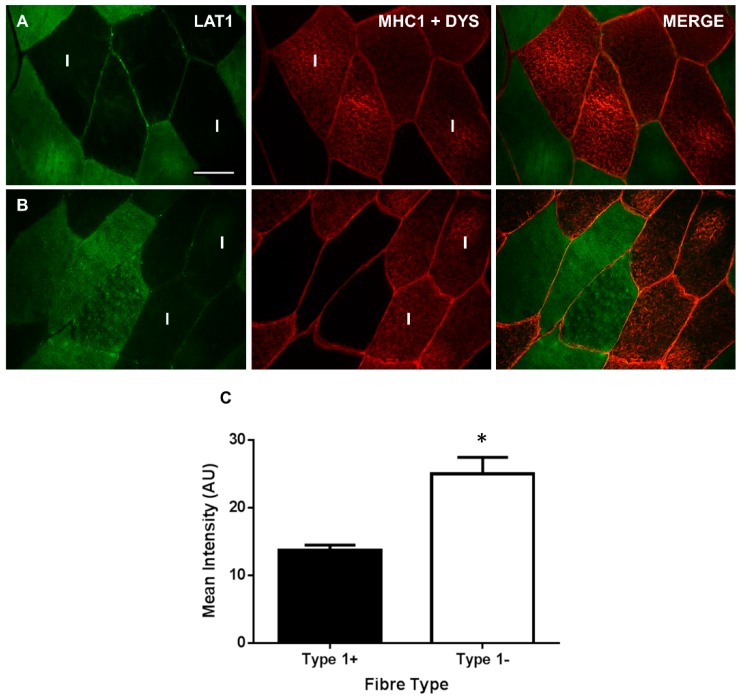
Immunofluorescent detection of LAT1 in human vastus lateralis muscle co-stained with myosin heavy chain 1 (MHC1) and dystrophin (DYS). Sections were stained with anti-LAT1 antibody (far left panels, green) and then co-stained with dystrophin (middle panels, red) for the identification of the sarcolemma and MHC1 (middle panels, red sarcoplasmic staining, marked with I) for the identification of type I muscle fibres. Representative images from two participants are displayed (**A**,**B**). Quantification of the mean immunofluorescent staining in type I and type II fibres displayed a greater staining in type II fibres (**C**). On average, 103 ± 17 fibres were quantified per participant. Values are Mean ± SE. *, significantly different to type I (*p* < 0.01). The scale bar in the top left panel is 50 µm and all images were captured at the same magnification.

To examine fibre type distribution, LAT1 was co-stained with myosin heavy-chain 1 (MHC1) to identify type I fibres. This allowed us to quantify LAT1 positive staining in type I and II fibres. Representative images of this stain (Figure 3A,B) displayed a difference in LAT1 staining between fibre types with a greater staining intensity seemingly apparent in type II fibres. Quantification of the staining intensity in each fibre type (Figure 3C) confirmed this as an 82.7% greater staining intensity was noted in type II fibres compared to type I fibres (25.07 ± 5.93 vs. 13.71 ± 1.98, *p* < 0.01, Figure 3C). This results suggests that a greater amount of LAT1 is expressed in more glycolytic fibres, which do not express MHC1; however, it seems the difference predominantly occurs in the sarcoplasm as positive LAT1 staining is still apparent at the sarcolemma of type I fibres (Figure 3A,B).

### 3.3. LAT1 Localises Close to the Microvasculature

**Figure 4:**
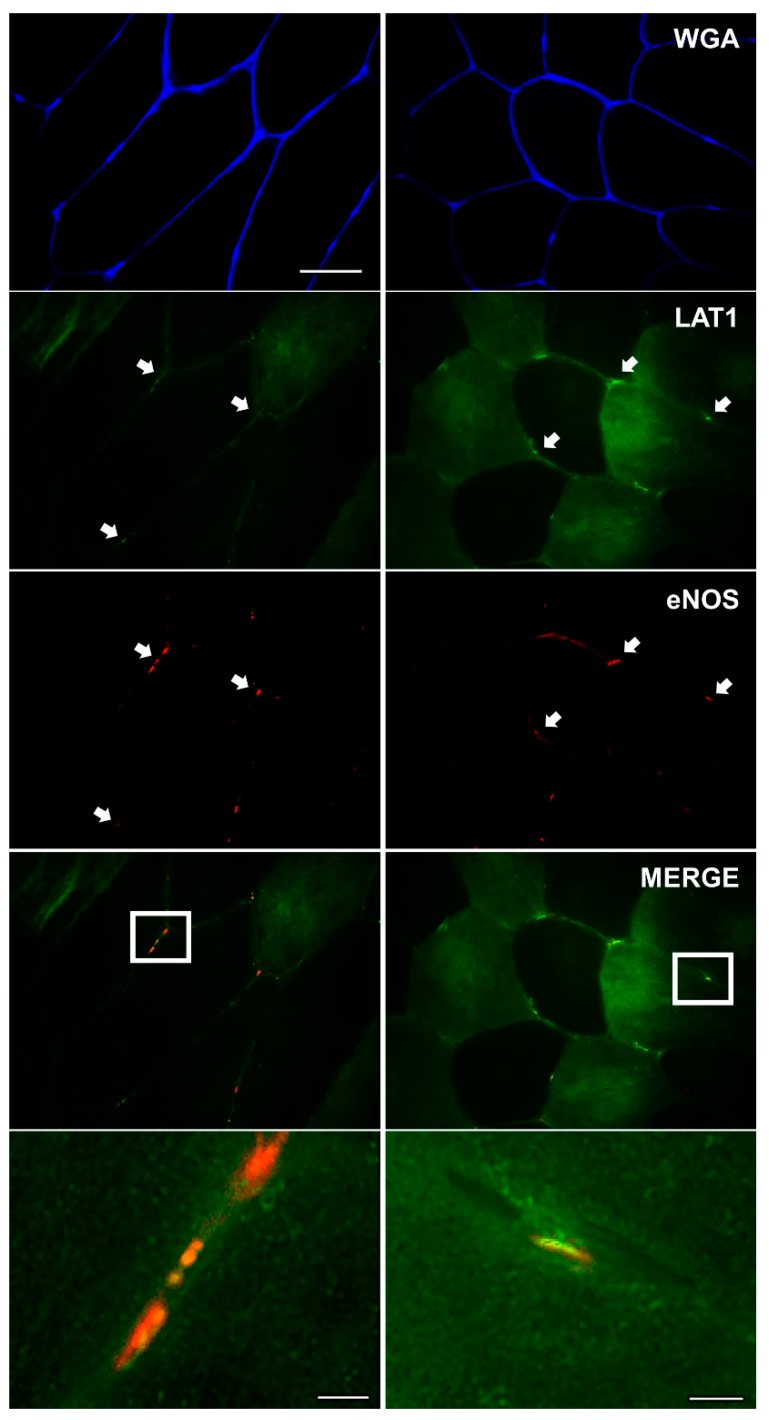
Immunofluorescent detection of LAT1 in human vastus lateralis muscle co-stained with endothelial nitric oxide synthase (eNOS) and Wheat Germ Agglutinin (WGA). Sections were stained with anti-LAT1 antibody (green) and then co-stained with eNOS (red) for the identification of blood vessels. WGA (blue) was used to identify membrane borders. Representative images from two participants are displayed. Merged images display positive LAT1 staining localising close to positive eNOS staining, shown in greater detail in zoomed images (bottom panels). Scale bar in top left panel is 50 µm and all images (except zoomed images) were captured at the same magnification. Scale bar in bottom panels is 5 µm.

To assess LAT1 interaction with the microvasculature, LAT1 was co-stained with anti-eNOS antibody, in order to identify blood vessels. This antibody has previously been utilised in human skeletal muscle to positively stain capillaries and other micro-vessels [30]. Here, it was apparent that positive LAT1 staining occurred close to positive staining of the microvasculature (eNOS), as shown in the merged and zoomed images (Figure 4, bottom panels), suggesting that LAT1 localises close to skeletal muscle vasculature at rest.

## 4. Discussion

LAT1/SLC7A5 is a transmembrane amino-acid transporter, which imports leucine and other BCAAs into cells in exchange for glutamine. This places LAT1 as a central regulator of skeletal muscle protein dynamics given the importance of leucine in activating mTORC1 and initiating MPS after feeding [31]. Previous studies investigating LAT1 in human skeletal muscle have focussed on gene expression and protein levels of this transporter in whole muscle homogenates, measures that do not provide much information about LAT1 physiological function as the LAT1 protein is only ‘active’ when associated with cellular membranes [9]. Therefore, in this study, we aimed to validate and optimise an immunofluorescence staining method to visualise LAT1 in human skeletal muscle. Using this approach, we observed LAT1 to be in two pools within in basal human skeletal muscle: within the sarcoplasm and associated with the sarcolemma. Furthermore, we report novel information showing LAT1 to be expressed in a fibre-specific manner and located in close proximity to the microvasculature.

Staining for LAT1 in fasted human skeletal muscle tissue displayed a strong immunoreactivity close to the sarcolemmal membrane (Figure 2), which was confirmed by its presence within a giant sarcolemmal vesicle preparation (Figure 2D). This fraction, which is primarily sarcolemmal membrane but may also contain recycling endosomal membranes [32], has been previously shown to contain intracellular fat (e.g., FAT/CD36) and glucose transporters (e.g., GLUT4) in rodent and human muscle [28,33]. This primary location of LAT1-positive staining along the sarcolemmal membrane is in congruence with its purported role as a transceptor [34]. As the primary transporter of BCAAs into muscle cells [19], fully functioning LAT1 would need to associate with the sarcolemmal membrane in order to convey free amino acids into muscle fibres [35]. Structurally, LAT1, a permease, can associate with a glycoprotein, CD98 (SLC3A2) [35,36], to form a heterodimer at a membrane. This association tethers LAT1 to the membrane, allowing the permease to transport amino acids across the membrane [36,37]. Furthermore, any role as a sensor of extracellular amino acids would also require LAT1 to be associated with the sarcolemma, as either a transporter or a signalling molecule LAT1 would have to associate with the membrane [8,34]. Although our data is the first study to utilise immunofluorescence staining techniques to detect LAT1 in human skeletal muscle, this protein has previously been visualised in other tissues. In human placenta, LAT1 was visualised predominantly at the microvillous membrane of terminal villi and was essential for leucine uptake into placenta cells [38]. Furthermore, LAT1 is located in plasma membranes of Laryngeal Squamous tumours [39], where amino acid uptake is elevated to provide substrates for tumour growth [40]. Several other tissues also express LAT1 principally in membranous structures including foetal intestines [41], non-small cell lung carcinomas [42] and ovarian tumours [43]. This consistent visualisation of LAT1 at membranes of tissues where protein synthesis/cell division is high consolidates our findings in human skeletal muscle, a tissue in which a regular supply of EAAs is imperative for optimal cell function and turnover [2].

Skeletal muscle is perfused by an intricate network of capillaries, and other microvessels, providing the working tissue with adequate supplies of oxygen and substrates whilst simultaneously removing waste products. Essential amino acids are transported to the muscle via this network, entering the bloodstream via absorption in the small intestine after food is consumed [44]. As this is the most ready supply of essential amino acids for skeletal muscle (other than intracellular amino acid recycling from protein breakdown), we investigated whether LAT1 would localise close to blood vessels. We display strong LAT1 immunoreactivity with eNOS. Such close association between an area of high supply (bloodstream) and one of high demand (skeletal muscle) suggests a high efficiency of amino acid transport in human skeletal muscle. Similar localisations have been displayed in other tissues with high LAT1 expression noted at blood–brain barriers in rodents [45,46] and chickens [47]. Additionally, other amino acid transporters are also seen in these regions [47], further implying a high efficiency of amino acid transport at blood–tissue junctions.

When staining for the LAT1 protein in skeletal muscle, a difference in the intensity of LAT1 staining between some fibres was apparent. We hypothesised that this may be specific to a particular fibre type and confirmed this by co-staining LAT1 with an anti-MHC1 antibody to identify type I (oxidative) fibres (Figure 3). LAT1 expression was greater in type II fibres and this seemed to be driven by a greater staining intensity within the sarcoplasm of these fibres, suggesting a greater dynamic pool in these fibres. This was confirmed via immunoblotting of mixed human skeletal muscle giant sarcolemmal vesicles and cytoplasmic fractions (Figure 2D), the latter of which may include membranes associated with sorting endosomes, lysosomes, and intracellular organelles (e.g., endoplasmic reticulum, Golgi apparatus) that would be distinct from those extracted in the giant sarcolemmal vesicles [32,48]. Immunoblotting displayed expression of the LAT1 protein in both fractions adding further validation to our observations of greater LAT1 staining intensity in the sarcoplasm of type II fibres. Several mechanisms within skeletal muscle may explain this apparent increase in sarcoplasmic LAT1 within more glycolytic fibres. Firstly, increases in LAT1 protein could result from mTORC1 activation. In response to essential amino acid ingestion [12], resistance exercise [11], or both in combination [10,49], stimuli that have been consistently reported to transiently activate mTORC1, LAT1 gene expression and protein levels are elevated in young and old males. In addition, alterations in LAT1 gene expression and protein levels following platelet-derived growth factor incubation are abolished by co-incubation with rapamycin [24], an mTORC1 inhibitor. As type II fibres show a greater elevation in mTORC1 activity in response to exercise [50], it is possible that this drives greater transcription/translation of LAT1 in these fibres, which remains apparent in fasted tissue. It may also be possible that, due to the greater levels of mTORC1 activity experienced in these fibres, LAT1 is required in the membrane of intracellular lysosomes as shown in some in vitro models [51]. Here, LAT1 could maintain intralysosomal amino acid concentrations, which are believed to then contribute to mTORC1 activation [52]. It is also possible that sarcoplasmic LAT1 may translocate toward the sarcolemmal membrane in response to stimuli where an increase in amino acid transport occurs (i.e., mechanical stimulation or feeding [20]). In fact, the translocation capacity of LAT1 has been previously reported in BeWo Chariocarcinoma cells after exposure to IGF-1, where the transporter translocated from perinuclear regions toward the cell membrane [53]. An increase in membrane-associated LAT1 protein will then increase the amount of amino acids that can be transported into the cell for protein synthesis/repair. Since we show a greater intracellular concentration of LAT1 in type II fibres, if LAT1 does translocate, this would suggest that type II fibres would have the greatest anabolic potential. It is important that this fibre-type difference in LAT1 expression is further investigated to fully understand the underpinning mechanisms and how signalling/metabolism in these fibres are affected.

## 5. Conclusions

To conclude, we have validated and optimised a method by which LAT1 can be visualised in human skeletal muscle. As this amino acid transporter is only active when associated with cellular membranes, this novel method allows a greater depth of investigation into the dynamics of LAT1 in human muscle in response to anabolic stimuli. For the first time, we have identified LAT1 to be primarily located close to the plasma membrane of all fibres and in close proximity to the microvasculature. Furthermore, we note a greater immunoreactivity of this protein in the sarcoplasm of type II fibres, potentially supporting the greater anabolic potential in these fibres. We believe this technique may provide a valuable insight into the role of LAT1 in skeletal muscle amino acid sensing, intracellular signalling, and subsequently adaptation. It is important to acknowledge, however, that the use of immunofluorescence microscopy as discussed here can only infer localization of target proteins. To gain greater insight into whether the LAT1 protein is directly localized to the sarcolemmal membrane, microvasculature or actin myosin bundles, immune-electron microscopy could be utilized and/or isolation of giant sarcolemmal vesicles. However, the present immunofluorescence technique requires less tissue (i.e., ~20 mg vs. >200 mg) than isolated giant sarcolemmal vesicles and can be characterized from frozen as compared to fresh samples, which represent significant advantages when muscle tissue samples are limited. Future research should now focus on identifying the possible underlying mechanisms, and consequences, of the fibre type differences reported here. Moreover, investigations into how factors such as ageing, exercise and feeding regulate LAT1 cellular location should also be undertaken. As LAT1 is the principal mechanism by which leucine enters the cell, approaches to increase LAT1 content or activity in skeletal muscle could have substantial relevance to post-exercise recovery strategies in which dietary protein or amino acids are of direct importance.

## Figures and Tables

**Table 1:** Summary of antibodies used.

Primary Antibody	Source	Dilution	Secondary Antibody	Dilution
Rabbit polyclonal anti-Solute Carrier family 7 member 5 (SLC7A5) antibody isotype IgG	Abcam, ab85226	1:100	Goat anti-rabbit IgG(H+L) Alexa^®^488	1:200
SLC7A5 peptide	Abcam, ab192836	1:10	N/A	N/A
Mouse monoclonal anti-Dystrophin antibody, isotype IgG2a	DSHB, MANDYS1 3B7	1:200	Goat anti-mouse IgG2a Alexa^®^594	1:200
Mouse monoclonal anti-Myosin Heavy Chain 1 (MHC1) antibody, isotype IgM	DSHB, A4.480	1:500	Goat anti-mouse IgM Alexa^®^594	1:200
Mouse monoclonal anti-endothelial nitrate oxide synthase (eNOS) antibody, isotype IgG1	BD Transduction, #610297	1:200	Goat anti-mouse IgG1 Alexa^®^594	1:200
Wheat Germ Agglutinin-350	W11263, Invitrogen	1:20	Alexa Fluor^®^ 350 Conjugated	N/A
